# Growth of Self-Catalyzed InAs/InSb Axial Heterostructured Nanowires: Experiment and Theory

**DOI:** 10.3390/nano10030494

**Published:** 2020-03-10

**Authors:** Omer Arif, Valentina Zannier, Vladimir G. Dubrovskii, Igor V. Shtrom, Francesca Rossi, Fabio Beltram, Lucia Sorba

**Affiliations:** 1 NEST, Istituto Nanoscienze—CNR and Scuola Normale Superiore, Piazza San Silvestro 12, I-56127 Pisa, Italy; omer.arif@sns.it (O.A.); fabio.beltram@sns.it (F.B.); lucia.sorba@nano.cnr.it (L.S.); 2 School of Photonics, ITMO University, Kronverkskiy pr. 49, 197101 St. Petersburg, Russia; dubrovskii@mail.ioffe.ru; 3 The Faculty of Physics, St. Petersburg State University, Universitetskaya Emb. 13B, 199034 St. Petersburg, Russia; i.shtorm@spbu.ru; 4 IMEM—CNR, Parco Area delle Scienze 37/A, I-43124 Parma, Italy

**Keywords:** InSb nanowires, axial heterostructures, self-catalyzed growth, modelling

## Abstract

The growth mechanisms of self-catalyzed InAs/InSb axial nanowire heterostructures are thoroughly investigated as a function of the In and Sb line pressures and growth time. Some interesting phenomena are observed and analyzed. In particular, the presence of In droplet on top of InSb segment is shown to be essential for forming axial heterostructures in the self-catalyzed vapor-liquid-solid mode. Axial versus radial growth rates of InSb segment are investigated under different growth conditions and described within a dedicated model containing no free parameters. It is shown that widening of InSb segment with respect to InAs stem is controlled by the vapor-solid growth on the nanowire sidewalls rather than by the droplet swelling. The In droplet can even shrink smaller than the nanowire facet under Sb-rich conditions. These results shed more light on the growth mechanisms of self-catalyzed heterostructures and give clear route for engineering the morphology of InAs/InSb axial nanowire heterostructures for different applications.

## 1. Introduction

InSb has the smallest band gap, the highest electron mobility and the largest thermo-power figure of merit among the entire family of III-V semiconductor compounds [1,2], which makes this material ideal for various applications in high speed electronics and photonics. Unfortunately, however, epitaxial growth of InSb in the form of two-dimensional layers is challenging due to its large lattice mismatch with common semiconductor substrates [3,4]. There have been many efforts to grow InSb in the form of nanowires (NWs) on both on InSb substrates [5] and on lattice-mismatched substrates such as Si and InAs [6,7,8,9,10,11,12,13], which enables a radical improvement of its crystalline quality and may pave new ways to fabricate InSb-based devices. Despite this progress, it is admittedly challenging to maintain the necessary control over the morphology and dimensions of InSb NWs, and many fundamental aspects of their growth and related properties are not yet fully understood. It is difficult to nucleate InSb NWs directly on a dissimilar substrate, which is why they are often grown on InAs NW stems [12]. Furthermore, the top InSb segment rapidly widens with respect to InAs stem [6,7,8,9,10,11,12,13]. Similar effect is observed for ternary InAsSb segments [14,15]. This radial extension of the InSb segment can be caused by several reasons, such as the droplet inflation with either In or Sb atoms, or a rapid radial growth on the NW sidewalls, but the exact mechanism has not been revealed to our knowledge. Most efforts were focused on the Au-assisted growth of InAs/InSb heterostructured NWs [6,8,9,11]. However, Au incorporation in NWs [16] may degrade their electrical and optical properties by creating deep levels that subsequently act as recombination centers for the charge carriers [17], even if Au contamination has not been detected in Au-assisted InSb NWs grown by MOVPE [5]. Furthermore, the use of Au is not compatible with CMOS processing. On the other hand, self-catalyzed [18,19,20], or catalyst-free [21,22,23], growth methods enable a homo system that avoids any possible risk of an Au seed. It has recently been demonstrated that catalyst-free growth through selective area epitaxy (SAE) of ternary InAs_1−x_Sb_x_ NWs on InAs stems cannot work for x higher than ~0.4 [24]. More Sb-rich alloys prefer to grow on the InAs sidewalls and the NWs finally become nano-discs. Catalyst-free growth of pure InSb in the form of InAs/InSb axial heterostructured NWs is hence extremely challenging. The only remaining way for the Au-free synthesis of InSb NWs on InAs stems should then be the self-catalyzed (or self-assisted) approach [25,26], in which the Au catalyst is replaced by In. To the best of our knowledge, only a few studies have been reported on self-catalyzed InAs/InSb NWs [7,27,28] and a detailed description of the growth mechanisms is still lacking. Consequently, here we present the first systematic analysis of the self-catalyzed growth of InAs/InSb axial heterostructured NWs on silicon substrates by Chemical Beam Epitaxy (CBE). Our investigation shed new light on some general features of the growth mechanisms and the resulting properties of NWs, including the evolution of the InSb morphology, crystal phase and the role of In droplets in forming InAs/InSb axial heterostructured NWs. Most importantly, this understanding allows for the realization of Au-free and CMOS-compatible InAs/InSb heterostructured nanostructures with well controlled properties.

## 2. Methods

InAs/InSb axial heterostructured NWs were grown on Si (111) substrates by CBE in a Compact-21 system (Riber, Paris, France). The metal-organic (MO) precursors used for growth were trimethylindium (TMIn), *tert*-butylarsine (TBAs), and *tert*-dimethyl-aminoantimony (TDMASb). In the first step, catalyst-free InAs NWs were grown via the vapor-solid (VS) growth mode. Details of the substrate preparation and the growth procedure can be found in [21]. The average length and edge-to-edge diameter of InAs stem were 460 ± 50 nm and 60 ± 10 nm, respectively, with no tapering from base to top. In the second step, InSb segments were grown on these InAs NW stems. In order to study in detail, the growth mechanisms of InSb segment, different TMIn (*F_In_*) and TDMASb (*F_Sb_*) line pressures and time durations were investigated at a fixed growth temperature of 430 ± 10 °C. At the end of growth, the TMIn flux was stopped and the sample was cooled down to 150 °C in 3 min, linearly decreasing the TDMASb line pressure to 0 Torr. The NW morphology was characterized by scanning electron microscopy (SEM) in a Merlin field emission microscope (Zeiss, Jena, Germany) operated at 5 KeV. For imaging the NWs were mechanically transferred from the as-grown substrates onto a Si substrate, in order to measure the geometrical parameters (nanoparticle (NP) height and base radius, InSb segment length and diameter) from a 90° projection. Crystal structure and chemical composition of the NWs were measured by transmission electron microscopy (TEM) using a JEM-2200FS microscope (JEOL, Tokyo, Japan) operated at 200 keV, equipped with an in-column Ω filter and a detector for X-ray energy dispersive spectroscopy (EDX). Imaging was performed in high resolution (HR) TEM mode combined with zero-loss energy filtering. For TEM characterization, the NWs were mechanically transferred to carbon-coated copper grids.

## 3. Results and Discussion

**Figure 1:**
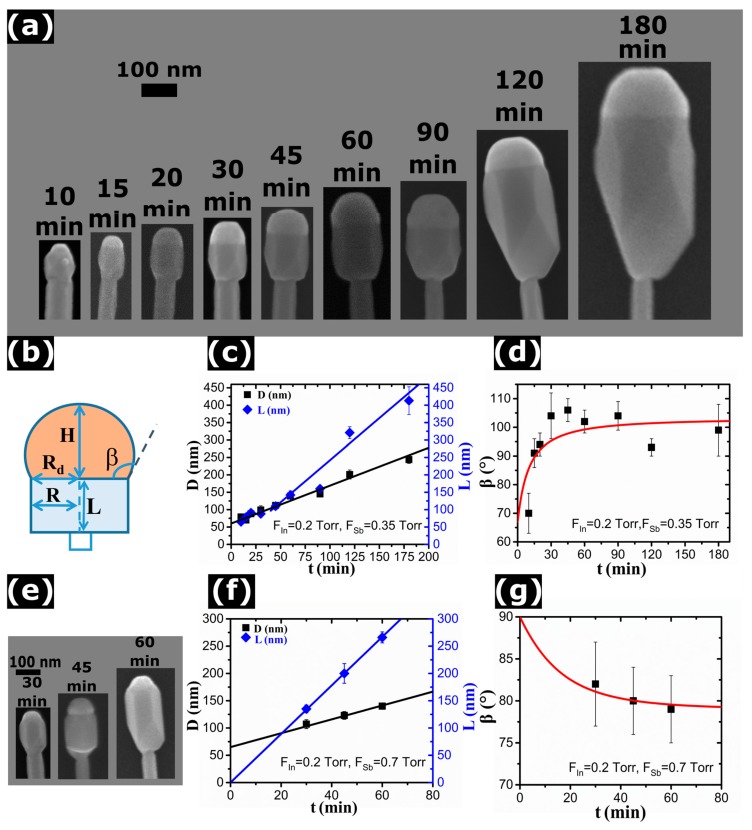
(**a**) Series of SEM images of InAs/InSb axial heterostructured NWs with In droplets on top, obtained with the line pressures *F_In_* = 0.2 Torr and *F_Sb_* = 0.35 Torr for different InSb growth times as indicated in each panel. The In droplet nucleates smaller than the NW facet, but then extends its base to cover the whole NW already after 15 min of InSb growth. (**b**) Schematic view of the measured geometrical parameters. (**c**) Time evolution of the diameter and length of InSb segments (symbols) and (**d**) time evolution of the contact angle of In droplets on top of InSb segments (symbols) of the sample series shown in (**a**). (**e**) Series of SEM images of InAs/InSb axial heterostructured NWs with In droplets on top, obtained under *F_In_* = 0.2 Torr and *F_Sb_* = 0.7 Torr for 30, 45, and 60 min of InSb growth time. The droplet diameter appears systematically smaller than the NW diameter. (**f**) Time evolution of the diameter and length of InSb segments (symbols) and (**g**) time evolution of the contact angle of In droplets on top of InSb segments (symbols) of the sample series shown in (**e**). The lines in (**c**,**d**,**f**,**g**) are theoretical fits discussed in the modeling section.

We first studied the evolution of the InSb segment as a function of its growth time t. In this series of samples, the line pressures *F_In_* and *F_Sb_* were fixed at 0.2 Torr and 0.35 Torr, respectively, while the growth times of InSb were varied (*t* = 10, 15, 20, 30, 45, 60, 120 and 180 min). SEM images of one representative NW from each sample are shown in Figure 1a. The InAs/InSb interface is always well visible thanks to a larger diameter of the InSb segment. We performed EDX analysis of the longest NWs (with *t* = 180 min). We did not find any Sb signal around the InAs stem and the InAs/InSb axial interface was quite sharp, corresponding to the position at which the NW diameter started to increase. Therefore, the InSb segment length can be measured directly from the SEM images as the distance from the InAs/InSb interface to the InSb/NP interface. A frozen In droplet (the NP) is always visible on top of InSb segment, clearly revealing the self-catalyzed VLS growth mechanism for InSb section. Accumulation of In on the NW top must be due to In-rich conditions during growth of InSb, as in Ref. [18,29], for In and Ga catalyzed InAs and GaAs NWs.

We measured the following parameters for each sample (averaged over ~30 NWs): the maximum diameter of InSb segment *D* = 2*R*, with *R* as the radius of the segment, the length of InSb segment *L*, the base radius *R_d_* and the height *H* of the NP, as described in panel (b) of Figure 1. All the average quantities with the standard deviation, for all the series of samples, are reported in the Supplementary material file. The time evolution of the NP shape under these growth conditions shows that it first appears smaller than the maximum InSb diameter due to tapering of the top NW section (after 10 min of InSb growth), but soon is pinned at the corners of vertical NW, with the aspect ratio (*H*/*R_d_*) increasing toward longer times. The InSb segment length *L* and diameter *D* versus time *t* are shown in panel (c). We can see that both quantities increase with the growth time, but the InSb length increases faster than the diameter. Both length and diameter are approximately linear in time. Assuming spherical cap shape of the NP resting on the NW top facet, the contact angle β can be obtained using the known expression tan(*β*/2) = *H*/*R_d_* [30]. This is a standard method of measuring the contact angle [31]. In order to verify that the droplet geometrical parameters measured ex-situ are representative of the actual droplet shape during growth, we carried out some cooling experiments with and without TDMASb flux (see the Supplementary Material for the details). The results confirm that the cooling down step does not affect the NW and droplet geometry, so the ex-situ measurements well reproduce the real shape and can safely be used for the *β* calculation. The plot of the contact angle versus time is shown in Figure 1d. It is seen that the contact angle increases quite rapidly at the beginning but then saturates at 102° ± 2°.

Next, we investigated the morphological evolution of InSb segments using a less In-rich condition by increasing the *F_Sb_* value from 0.35 Torr to 0.7 Torr and keeping the same *F_In_* of 0.2 Torr. We grew three samples with *t* = 30, 45 and 60 min, for which the representative SEM images are shown in Figure 1e. Figure 1f shows the measured diameter and length of InSb segments as a function of the growth time. It is seen that the droplet is always smaller than the maximum NW diameter due to tapering of the NW top. Furthermore, the droplet diameter stays almost constant during growth, while the maximum InSb diameter increases linearly with time according to Figure 1f. By measuring the aspect ratio of the NPs, we deduced their contact angle plotted in Figure 1g. It is seen that for these growth conditions, the contact angle saturates at approximately 79°.

**Figure 2:**
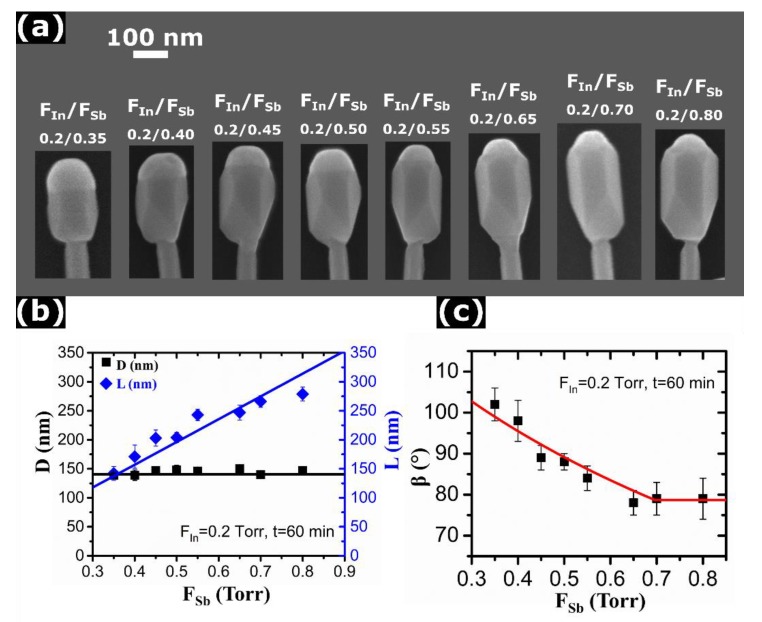
(**a**) Series of SEM images of InAs/InSb NWs, obtained after 60 min of InSb growth at a fixed *F_In_* of 0.2 Torr and different *F_Sb_*, yielding different In/Sb line pressure ratios as indicated in each panel. The droplets become smaller than the NW facet for higher *F_Sb_*. (**b**) Diameter and length of InSb segment versus the *F_Sb_* (symbols). (**c**) Contact angle of In droplets on top of InSb segments versus *F_Sb_* (symbols). The lines in (**b**,**c**) are theoretical fits discussed in the modeling part. The change of the slope in the model fit comes from the minimum stable contact angle of 79°.

By varying *F_Sb_* and *F_In_* separately, we studied the effect of the In/Sb line pressure ratio on the morphology of InSb segments. Figure 2a shows the representative SEM images of a series of InAs/InSb NWs as a function of *F_Sb_*, obtained by keeping *F_In_* at 0.2 Torr and varying *F_Sb_* from 0.35 Torr to 0.80 Torr. The InSb growth time was 60 min for all samples. It is clearly seen that the size of In droplets decreases and the length of InSb segment increases with increasing the TDMASb line pressure. For lower TDMASb pressures (*F_Sb_* < 0.55 Torr), the droplet covers the whole top facet of InSb NW, while for higher TDMASb line pressures it becomes smaller than the facet. The maximum *F_Sb_* at which the In droplet is preserved on the NW top equals 0.8 Torr. Higher TDMASb line pressure leads to a transition from the VLS growth to the catalyst-free vapor-solid (VS) mode, where no axial growth of InSb is observed. Instead, InSb starts forming a shell around the InAs stem (see the Supplementary material). We can thus conclude that axial growth of InSb on InAs can only proceed in the presence of an In droplet through the VLS growth mode, while no axial growth occurs in the catalyst-free VS regime, as observed earlier in [14,15]. Figure 2b shows the diameter and length of InSb segment as a function of *F_Sb_*. It is seen that, while the In droplet size gradually decreases with increasing the TDMASb line pressure, the diameter of InSb segment remains constant. It clearly demonstrates that radial growth of InSb depends neither on the TDMASb line pressure nor on the In droplet size. Furthermore, the radial growth rate remains the same regardless of NW tapering at the top. Hence, radial growth should proceed independently of the VLS process occurring on the NW top. The length of InSb segment increases almost linearly with *F_Sb_*, as usually observed in self-catalyzed III-V NWs [25,29,32]. By applying the same method as above, we deduced the droplet contact angle as a function of *F_Sb_*, shown in Figure 2c. The contact angle gradually decreases with increasing the TDMASb line pressure. For lower pressures (*F_Sb_* < 0.4 Torr), it remains larger than 90°, while for higher pressures (above ~0.65 Torr) it saturates at ~79°. The droplet volume further decreases by shrinking its base diameter smaller than the facet.

**Figure 3:**
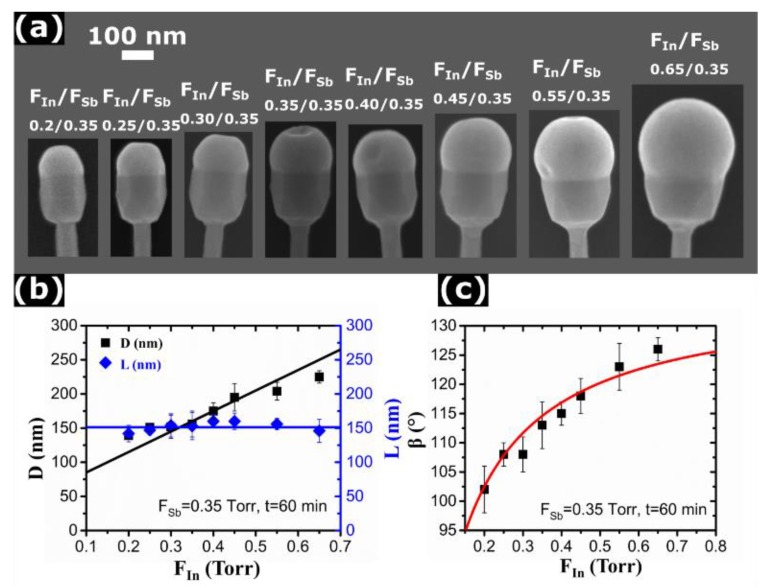
(**a**) Series of SEM images of InAs/InSb axial heterostructured NWs with In droplets on top, obtained after 60 min of InSb growth at a fixed *F_Sb_* of 0.35 Torr and different *F_In_*, yielding different Sb/In line pressure ratios indicated in each panel. Under these highly In-rich conditions, the In droplets always cover the whole NW facet. (**b**) Diameter and length of InSb segments versus the *F_In_* (symbols). (**c**) Contact angle of In droplets on top of InSb segments versus *F_In_* (symbols). The lines in (**b**,**c**) are theoretical fits discussed in the modeling section.

Figure 3a shows a series of SEM images of InAs/InSb NWs obtained by varying *F_In_* from 0.2 Torr to 0.65 Torr at a fixed *F_Sb_* of 0.35 Torr. The InSb growth time was fixed to 60 min for all samples. Clearly, all these growths proceed under highly In-rich conditions, where the volume of the In droplet gradually increases with increasing *F_In_*. The In droplets cover the whole NW top facets in all cases. Figure 3b shows the InSb diameter and length versus *F_In_*. The diameter increases linearly with *F_In_*, while the length is independent of *F_In_*. We can thus conclude that the axial growth rate of InSb segment is independent of *F_In_*, while the radial growth rate is proportional to *F_In_*. Figure 3c quantifies the droplet contact angle as a function of *F_In_*, showing a rapid increase at the beginning but then showing a tendency for saturating at around 125°. Further increase of the In droplet volume occurs by increasing the base radius.

**Figure 4:**
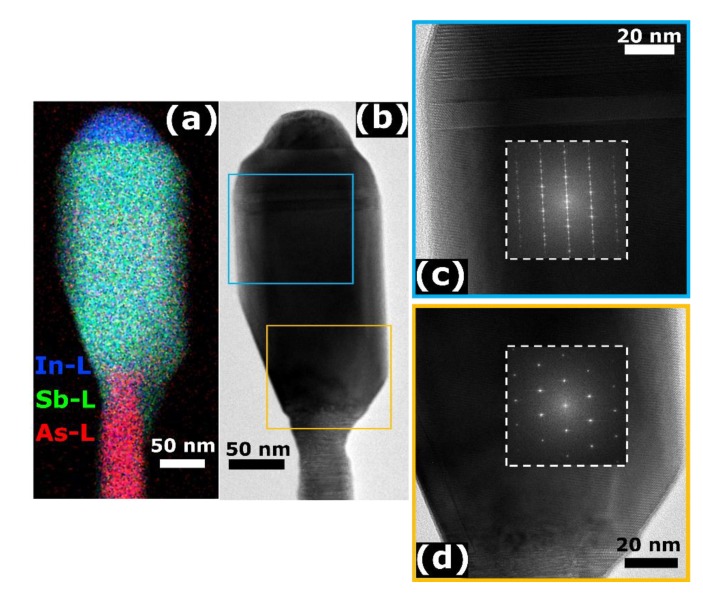
TEM analyses of the InAs/InSb NWs grown with *F_In_* = 0.2 Torr and *F_Sb_* = 0.7 Torr for 60 min. (**a**) EDX compositional map of a NW in which is visible the InAs stem in pink color, the InSb segment in green color and the In NP in blue. (**b**–**d**) show HR-TEM images of another NW with the FFTs of the selected portions (insets).

We performed TEM and EDX analyses of the InAs/InSb NWs grown under different conditions and obtained very similar results. Figure 4 shows two representative NWs from the sample grown for 60 min at *F_In_* = 0.2 Torr and *F_Sb_* = 0.7 Torr (additional TEM images of a sample grown using different growth conditions are provided in the Supplementary Material). Panel (a) shows the EDX map of a NW, while panel (b) is a HR-TEM image of another NW. Panels (c) and (d) are the magnified views of the selected portions of the NW framed by the colored squares in (b), with the insets showing the Fast Fourier Transforms (FFT) of the InSb lattice. In all the NWs analyzed, we found that the catalyst nanoparticles contain only In. The Sb concentration is always lower than 1%, regardless of the In/Sb precursor ratios used. From the HR-TEM and the FFT analyses, we found that the InAs stems have a mixed wurtzite/zincblende (WZ/ZB) crystal structure, while the InSb segments have the ZB structure with a few stacking faults, often followed by a thin WZ insertion at the NW top (close to the NW/NP interface). The TEM results confirm the self-catalyzed growth mechanism with pure In droplet on the NW top, as reported earlier in Refs. [12] and [24], and the good stability of the ZB crystal phase in InSb NWs regardless of the growth parameters employed. A more detailed discussion of this stability based on the surface energy considerations is given in the Supplementary material.

Let us now analyze the most important trends observed and present a model to explain and quantify these findings. For the fixed pyrolysis efficiencies at a given growth temperature, the atomic In and Sb fluxes entering the droplet should be proportional to the TMIn and TDMASb line pressures. This central assumption can be justified by the following facts. Our data clearly show that the axial growth rate is approximately proportional to *F_Sb_*, as demonstrated in Figure 2b. This is standard for self-catalyzed VLS growth [25,32,33,34,35] and occurs because the catalyst droplet serves as a reservoir of group III atoms (In in our case) and the VLS growth conditions are always group V limited. Including the re-emitted flux of group V atoms scattered from the substrate surface or the neighboring NWs [32] does not change the linear scaling of the axial growth rate with group V flux. On the other hand, group V species are not diffusive on the NW sidewalls [32,33,34]. Conversely, VS growth on the NW sidewalls is usually group III limited [15,26,33,36] and may involve surface diffusion of In adatoms. This would not change the linear scaling of the average NW diameter with group III flux, clearly demonstrated in Figure 3b. Diffusivity of In on Si(111) surface might be high, but we consider growth of InSb on InAs at a distance ~500 nm from the substrate. Surface diffusion of In is known to not strongly affect even the Au-catalyzed CBE growth of InAs NWs, which is evidenced by their Poissonian length distributions [37]. When the VLS growth is driven by surface diffusion, the NW length distributions become much broader, with the variance scaling as the squared mean length [38] (against the linear scaling in the Poissonian case [37]). Finally, we are dealing only with the average values of the InSb segment length, diameter, and In droplet angle. In this case, it seems reasonable to assume a linear scaling of the growth rates with the corresponding fluxes, leaving aside more delicate effects of re-emission, surface diffusion and random nucleation of NWs on the surface [37]. Deviations from the linear fits, seen in Figure 1c, Figure 2b and Figure 3b, might be due to the effects listed above.

**Figure 5:**
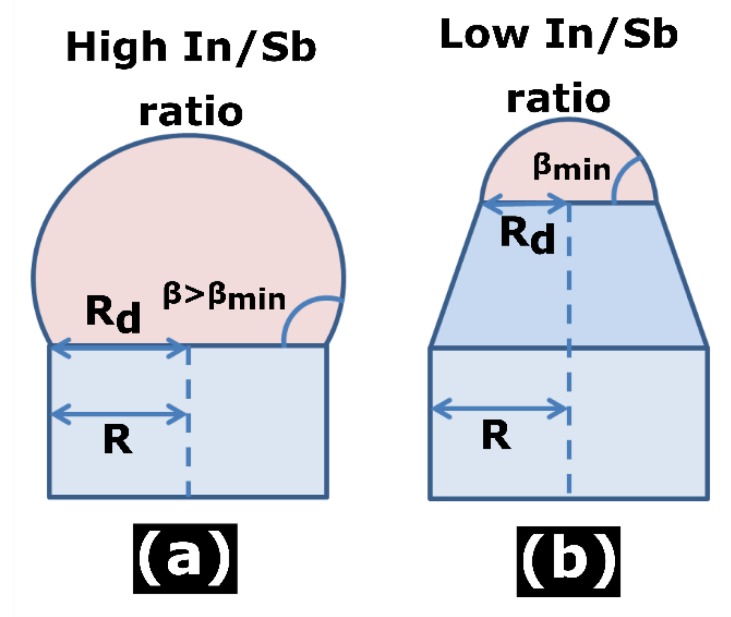
Illustration of (**a**) vertical (for *β* > *β*_min_) or (**b**) tapered (at *β* = *β*_min_) NW geometry, with *β*_min_ ≅ 79° as the small stable angle determined by the surface energetics.

We have seen that the In droplets cover the whole top facets of InSb NWs at any In/Sb ratio. However, higher In/Sb ratios yield vertical or even slightly inverse-tapered InSb NWs, such as shown in Figure 1a and Figure 3a, whereas lower In/Sb ratios lead to tapered NW tops, such as seen in Figure 2a and Figure 4a,b. Tapered NWs maintain a fixed contact angle of In droplet of ~79° until the transition from the VLS to VS growth regime at *F_In_*/*F_Sb_* = 0.2/0.9. The two typical geometries are illustrated in Figure 5 and can be understood on surface energetic grounds similarly to other III-V NWs.

In situ [31] and ex-situ [33] growth studies of self-catalyzed GaAs NWs show the bistability of the Ga droplet angle, with the two stable angles around 90° and 130°. Inside this range, NWs grow with vertical sidewalls, while they taper at the small or inverse-taper at the large stable contact angle. Similar behavior is observed for self-catalyzed GaP NWs, with the vertical growth region shrinking to a narrow range around 123° [35]. We have determined the small stable contact angle of In droplets ~79°. The large stable contact angle is around 125–130° according to Figure 3c. Clearly, the large stable contact angle corresponds to very high In/Sb ratios and is rarely reached in our experiments. Conversely, the small stable contact angle is systematically observed, and determines the minimum In/Sb ratio at which InSb NWs can be grown in the VLS mode. Therefore, the two geometries shown in Figure 5 will be used for modeling.

The VLS growth on the NW top occurring at the liquid-solid interface must be faster than the VS growth without any droplet. Therefore, the axial growth of InSb is mediated by nucleation of InSb islands under the droplet, even if it is smaller than the maximum NW diameter. This explains why maintaining the droplet is crucial for the formation of axial InAs/InSb NW heterostructures. Without any droplet, InSb tends to surround the InAs stem and forms a core-shell structure [15].

As discussed above, InSb segments grown on top of the InAs stems show the maximum radius increasing linearly with time, as demonstrated by Figure 1c,f. The measured content of Sb in the droplet is always negligible, and hence the droplet volume is controlled by the amount of In rather than Sb. The radial growth rate of InSb is independent of the TDMASb line pressure (Figure 2b) and it is proportional to the TMIn line pressure (Figure 3b). It is remarkable that the radial growth rate is exactly identical for different contact angles of the In droplets and even the NW configurations (vertical or tapered). Linear fits for the time and *F_In_* dependences of the In-limited radial growth rates of InSb, shown in Figure 1c,f and Figure 3b, respectively, yield:

(1) $$\frac{dR}{dt}=a_{In}F_{In}$$

with $a_{In}F_{In}=$ 0.57 ± 0.07 nm/min at *F_In_* = 0.2 Torr. Integration gives $R=R_{0}+a_{In}F_{In}t$, with *R*_0_ ≈ 30 ± 5 nm as the initial radius of InAs stems. This matches exactly the horizontal line in Figure 2b at *t* = 60 min. Therefore, radial growth of InSb segments proceeds in the VS mode and has nothing to do with the droplet size evolution, in sharp contrast with Refs. [29], [34], and [39].

On the contrary, the axial growth rate of InSb segments is independent of the TMIn line pressure and is proportional to the TDMASb line pressure (Figure 2b). According to Figure 1c,f, the NW length becomes a linear function of time after 30 min of growth. For shorter growth times, instead, the increase of length with t is slightly super-linear. This could be an effect of an overestimation of the InSb length at the beginning of the growth because of the InSb deposition on the tilted facets of the InAs stem NW tip, instead of the flat (111) top-facet (see Supplementary Material for more details). Furthermore, geometrical effects in directional CBE technique [40], may affect the early stage growth rate. Generally, the axial growth rate of self-catalyzed III-V NWs is proportional to the group V flux [25,26,29,32,33,34,39], however, the slope may depend on the droplet contact angle. Therefore, we can write:

(2) $$\frac{dL}{dt}=b_{Sb}\left(\beta \right)F_{Sb}$$

with a *β*-dependent *b_Sb_* in the general case (measured in nm/min × Torr). After the contact angle saturates to a certain *β_c_*, as in Figure 1d, the axial growth rate becomes independent of *β* and hence on the In flux. In particular, from the linear fits shown in Figure 1c,f, the axial growth rate is almost precisely doubled (4.5 ± 0.2 nm/min against 2.4 ± 0.12 nm/min) by increasing *F_Sb_* from 0.35 Torr to 0.7 Torr.

In order to describe the evolution of the droplet shape as a function of time and material fluxes, we note that the contact angle changes due to the two independent processes. (1) On one hand, the contact angle of a droplet pinned at the NW corners decreases due to the radial growth on the NW sidewalls. We use the circular geometry for the NW top facet and spherical cup droplet shape. Then, whenever the NW radius increases by *dR*, the β decreases by $d\beta =-f\left(\beta \right){\left(1+cos\beta \right)}^{2}dR/R$, with f(β)=(1−cosβ)(2+cosβ)/[(1+cosβ)sinβ] as the geometrical function relating the droplet volume V to the cube of its base according to $V=\left(\pi R^{3}/3\right)f\left(\beta \right)$ [41]. Using Equation (1), this gives ${\left(d\beta /dt\right)}_{1}=-f\left(\beta \right){\left(1+cos\beta \right)}^{2}a_{In}F_{In}/R$. (2) On the other hand, the contact angle changes due to any unbalanced In income from vapor and its sink due to the VLS growth of InSb NW section. In atoms should not desorb either from the droplet or the NW surface at 430 °C. Assuming also the absence of surface diffusion of In adatoms on the NW sidewalls [13,37], the total number of In atoms in the droplet $N_{In}$ changes according to $dN_{In}/dt=\left(\pi R^{2}/\Omega_{InSb}\right)\left[b_{In}\left(\beta \right)F_{In}-b_{Sb}\left(\beta \right)F_{Sb}\right]$. Here, $b_{In}(\beta )$ is a *β*-dependent adsorption coefficient on the droplet surface for In similar to the one in Equation (2) for Sb, and $\Omega_{InSb}=$ 0.0680 nm^3^ is the elementary volume of InSb pair in ZB InSb [36]. Since the droplet contains only In atoms, we can write the corresponding change in the droplet volume, which equals $\Omega_{In}N_{In}$ (where $\Omega_{In}=$ 0.0261 nm^3^ is the elementary volume of liquid In) [36]. At a fixed R, we can present the volume change solely through dβ according to $dN_{In}/dt=\left(\pi R^{3}/\Omega_{In}\right){\left(1+cos\beta \right)}^{-2}{\left(d\beta /dt\right)}_{2}$ [41].

Equating these two expressions, we obtain the contact angle change due to the In/Sb influx imbalance in the form ${\left(d\beta /dt\right)}_{2}=\left(\Omega_{In}/\Omega_{InSb}\right){\left(1+cos\beta \right)}^{2}\left[b_{In}\left(\beta \right)F_{In}-b_{Sb}\left(\beta \right)F_{Sb}\right]/R$. Therefore, the total change of the contact angle is given by:

(3) $$\frac{d\beta }{dt}=\frac{{\left(1+cos\beta \right)}^{2}}{R_{0}+a_{In}F_{In}t}\left[\frac{\Omega_{In}}{\Omega_{InSb}}\left(b_{In}\left(\beta \right)F_{In}-b_{Sb}\left(\beta \right)F_{Sb}\right)-f\left(\beta \right)a_{In}F_{In}\right]$$

where we use the result of integration of Equation (1) for *R* in the denominator. Here, the first bracket term stands for the droplet shape evolution in the VLS process, similar to [42], while the second describes the decrease of the contact angle by In-limited VS radial growth on the NW sidewalls.

The stationary contact angle *β_c_* is obtained from:

(4) $$\frac{\Omega_{In}}{\Omega_{InSb}}\left(b_{In}\left(\beta_{c}\right)F_{In}-b_{Sb}\left(\beta_{c}\right)F_{Sb}\right)=f\left(\beta_{c}\right)a_{In}F_{In}$$

provided that the left-hand side is positive. Of course, the small stable angle should be put to $\beta_{\min}$ if the solution to Equation (4) is smaller than $\beta_{\min}$, corresponding to the NW tapering as described above. Using the linear fits in Figure 1d at $\beta_{c}=\beta_{*}≅$ 102°, we obtain the unknown $b_{In}(\beta_{*})F_{In}^{*}$, corresponding to these particular stationary contact angle and material fluxes. It is remarkable that our experimental data allow for the determination of the complete set of parameters describing the morphological evolution under these growth conditions, summarized in Table 1.

Equation (3) describes the time evolution of the contact angle toward the stationary value determined by Equation (4). To simplify the analysis, we can expand the geometrical functions entering these equations in $\beta -\beta_{*}$ and keep only the linear terms: $b_{k}\left(\beta \right)≅b_{k}\left(\beta_{*}\right)+b_{k}^{\prime }\left(\beta_{*}\right)\left(\beta -\beta_{*}\right)$ for k= In, Sb, and $f\left(\beta \right)≅f\left(\beta_{*}\right)+f^{\prime }\left(\beta_{*}\right)\left(\beta -\beta_{*}\right)$. Additionally, we account for a decrease of ${\left(1+cos\beta \right)}^{2}$ in Equation (3) with increasing β by using a linear approximation. In the CBE system, beam angles of In and Sb with respect to the vertical equal to 38°. This allows us to find the constant $A=b_{In}^{\prime }\left(\beta_{*}\right)/b_{In}\left(\beta_{*}\right)=b_{Sb}^{\prime }\left(\beta_{*}\right)/b_{Sb}\left(\beta_{*}\right)≅$ 0.534 using the expressions for the geometrical factors of Ref. [40]. The final expressions for the droplet shape evolution contain no free parameters. The stationary contact angle is given by:

(5) $$\beta_{c}=\beta_{*}+\frac{0.84\left(x-y\right)}{\left(1.45x+0.84y\right)}$$

for $\beta_{c}\geq \beta_{\min}$ and $\beta_{c}=\beta_{\min}$ otherwise, with $x=F_{In}/F_{In}^{*}$, $y=F_{Sb}/F_{Sb}^{*}$. The time evolution to this stationary state is described by:

(6) $$\beta \left(t\right)=\frac{\beta_{c}\left(\beta_{max}-\beta_{0}\right)-\beta_{max}\left(\beta_{c}-\beta_{0}\right){\left(1+t/\tau \right)}^{-\delta }}{\beta_{max}-\beta_{0}-\left(\beta_{c}-\beta_{0}\right){\left(1+t/\tau \right)}^{-\delta }}$$

where:

$$\delta =4\left(\beta_{max}-\beta_{c}\right)\frac{\left(1.45x+0.45y\right)}{x},\;\tau =\frac{2R_{0}}{x}$$

and $\beta_{0}$ is the initial contact angle. Here, $\beta_{max}$ = 119° for $\beta_{c}=\beta_{*}=$ 102° at x=y=1. We take $R_{0}=30\pm 5$ nm and $\beta_{0}=82\pm 3^{0}$ to fit all the data shown in Figure 1, Figure 2, Figure 3 and Figure 4.

Without any free parameters, our model provides excellent fit to all the data. This includes linear time dependences of the radius and length of InSb sections, linear scaling of R with the In line pressure and L with the Sb line pressure, non-linear time evolution of the contact angle to a flux-dependent stationary values (Figure 1d,g), increase of the stationary contact angle with the In flux (Figure 3c) and its decrease with the Sb flux (Figure 2c), and the NW tapering at the small stable contact angle. Overall, the achieved quantitative correlation of the model with the data is quite remarkable.

## 4. Conclusions

In conclusion, our study of self-catalyzed InAs/InSb axial heterostructured NWs reveals some general trends, which should pertain to a wide range of epitaxy techniques and growth conditions. We have demonstrated that rapid radial growth of InSb is not a consequence of the droplet inflation but is rather due to the VS radial growth on the NW sidewalls. The presence of an In droplet on the NW top is absolutely required to maintain the axial growth of heterostructure. The growth kinetics of InSb NW sections as well as In droplets on their tops have been explained and quantified within a model containing no free parameters. More complex kinetic effects and local fluctuations of the material fluxes requires a separate study.

In-catalyzed VLS growth of different In-V NWs has been known before, along with the obvious fact that it is limited by group V flux. Widening of InSb section with respect to InAs stem is also a general phenomenon. However, it was not clear if this process was due to the droplet inflation or radial VS growth on the InSb sidewalls. Here, we have demonstrated that the widening is controlled by the VS growth on the sidewalls, so that In droplet may shrink and the whole NW extend. Most importantly, we have established a model for the InSb morphology, which uses only the experimentally extracted parameters and fits quite well all the data in a wide range of In/Sb ratios. While the growth geometry and critical values of the material fluxes may depend on a particular deposition technique, the obtained results should be useful for engineering the morphology and the resulting properties of Au-free InAs/InSb axial heterostructured NWs for different applications.

## Figures and Tables

**Table 1:** Parameters describing the morphological evolution of InAs/InSb NWs and In droplets under *F_In_* = 0.2 Torr and *F_Sb_* = 0.35 Torr.

$\beta_{*}$	$F_{In}^{*}$	$F_{Sb}^{*}$	$a_{In}F_{In}^{*}$	$b_{Sb}(\beta_{*})F_{Sb}^{*}$	$b_{In}(\beta_{*})F_{In}^{*}$	$\Omega_{In}/\Omega_{InSb}$	A
Degree	Torr	Torr	nm/min	nm/min	nm/min		
102 ± 2	0.2	0.35	0.57 ± 0.07	2.4 ± 0.12	6.18 ± 0.05	0.384	0.534

## Supplementary Materials

The following are available online at https://www.mdpi.com/2079-4991/10/3/494/s1. S1. Measured parameters of the NWs of all sample series, S2. Vapor-solid growth of InSb, S3. High-resolution transmission electron microscopy analysis, S4. X-ray energy dispersive spectroscopy data on the catalyst droplet, S5. ZB crystal phase of InSb segments, S6. Cooling down experiment, S7. InSb length at short growth times.

## References

[1] Vurgaftman I., Meyer J.R., Ram-Mohan L.R. (2001). Band Parameters for III-V Compound Semiconductors and Their Alloys. J. Appl. Phys..

[2] Caroff P., Wagner J.B., Dick K.A., Nilsson H.A., Jeppsson M., Deppert K., Samuelson L., Wallenberg L.R., Wernersson L.E. (2008). High-Quality InAs/InSb Nanowire Heterostructures Grown by Metal-Organic Vapor-Phase Epitaxy. Small.

[3] Capper P., Irvine S., Joyce T., Kasap S., Capper P. (2017). Epitaxial Crystal Growth: Methods and Materials. Springer Handbook of Electronic and Photonic Materials.

[4] Kanisawa K., Yamaguchi H., Hirayama Y. (2000). Two-Dimensional Growth of InSb Thin Films on GaAs(111)A Substrates. Appl. Phys. Lett..

[5] Badawy G., Gazibegovic S., Borsoi F., Heedt S., Wang C., Koelling S., Verheijen M.A., Kouwenhoven L.P., Bakkers E.P.A.M. (2019). High Mobility Stemless InSb Nanowires. Nano Lett..

[6] Webb J.L., Knutsson J., Hjort M., Gorji Ghalamestani S., Dick K.A., Timm R., Mikkelsen A. (2015). Electrical and Surface Properties of InAs/InSb Nanowires Cleaned by Atomic Hydrogen. Nano Lett..

[7] Anandan D., Kakkerla R.K., Yu H.W., Ko H.L., Nagarajan V., Singh S.K., Lee C.T., Chang E.Y. (2019). Growth of Foreign-Catalyst-Free Vertical InAs/InSb Heterostructure Nanowires on Si (111) Substrate by MOCVD. J. Cryst. Growth.

[8] Caroff P., Messing M.E., Mattias Borg B., Dick K.A., Deppert K., Wernersson L.E. (2009). InSb Heterostructure Nanowires: MOVPE Growth under Extreme Lattice Mismatch. Nanotechnology.

[9] Li A., Sibirev N.V., Ercolani D., Dubrovskii V.G., Sorba L. (2013). Readsorption Assisted Growth of InAs/InSb Heterostructured Nanowire Arrays. Cryst. Growth Des..

[10] Nilsson H.A., Caroff P., Thelander C., Larsson M., Wagner J.B., Wernersson L.E., Samuelson L., Xu H.Q. (2009). Giant, Level-Dependent g Factors in InSb Nanowire Quantum Dots. Nano Lett..

[11] Lugani L., Ercolani D., Beltram F., Sorba L. (2011). Growth Mechanism of InAs-InSb Heterostructured Nanowires Grown by Chemical Beam Epitaxy. J. Cryst. Growth.

[12] Li T., Gao L., Lei W., Guo L., Pan H., Yang T., Chen Y., Wang Z. (2013). InAs-Mediated Growth of Vertical InSb Nanowires on Si Substrates. Nanoscale Res. Lett..

[13] Lugani L., Ercolani D., Sorba L., Sibirev N.V., Timofeeva M.A., Dubrovskii V.G. (2012). Modeling of InAs-InSb Nanowires Grown by Au-Assisted Chemical Beam Epitaxy. Nanotechnology.

[14] Robson M., Azizur-Rahman K.M., Parent D., Wojdylo P., Thompson D.A., Lapierre R.R. (2017). Multispectral Absorptance from Large-Diameter InAsSb Nanowire Arrays in a Single Epitaxial Growth on Silicon. Nano Futur..

[15] Sokolovskii A.S., Robson M.T., Lapierre R.R., Dubrovskii V.G. (2019). Modeling Selective-Area Growth of InAsSb Nanowires. Nanotechnology.

[16] Perea D.E., Allen J.E., May S.J., Wessels B.W., Seidman D.N., Lauhon L.J. (2006). Three-Dimensional Nanoscale Composition Mapping of Semiconductor Nanowires. Nano Lett..

[17] Zhang G., Tateno K., Gotoh H., Sogawa T. (2012). Vertically Aligned InP Nanowires Grown via the Self-Assisted Vapor-Liquid-Solid Mode. Appl. Phys. Express.

[18] Gomes U.P., Ercolani D., Zannier V., David J., Gemmi M., Beltram F., Sorba L. (2016). Nucleation and Growth Mechanism of Self-Catalyzed InAs Nanowires on Silicon. Nanotechnology.

[19] Ambrosini S., Fanetti M., Grillo V., Franciosi A., Rubini S. (2011). Self-Catalyzed GaAs Nanowire Growth on Si-Treated GaAs(100) Substrates. J. Appl. Phys..

[20] Munshi A.M., Dheeraj D.L., Todorovic J., Van Helvoort A.T.J., Weman H., Fimland B.O. (2013). Crystal Phase Engineering in Self-Catalyzed GaAs and GaAs/GaAsSb Nanowires Grown on Si(111). J. Cryst. Growth.

[21] Gomes U.P., Ercolani D., Sibirev N.V., Gemmi M., Dubrovskii V.G., Beltram F., Sorba L. (2015). Catalyst-Free Growth of InAs Nanowires on Si (111) by CBE. Nanotechnology.

[22] Soo M.T., Zheng K., Gao Q., Tan H.H., Jagadish C., Zou J. (2016). Growth of Catalyst-Free Epitaxial InAs Nanowires on Si Wafers Using Metallic Masks. Nano Lett..

[23] Dimakis E., Lähnemann J., Jahn U., Breuer S., Hilse M., Geelhaar L., Riechert H. (2011). Self-Assisted Nucleation and Vapor-Solid Growth of InAs Nanowires on Bare Si(111). Cryst. Growth Des..

[24] Yip S., Shen L., Ho J.C. (2019). Recent Advances in III-Sb Nanowires: From Synthesis to Applications. Nanotechnology.

[25] Colombo C., Spirkoska D., Frimmer M., Abstreiter G., Fontcuberta I., Morral A. (2008). Ga-Assisted Catalyst-Free Growth Mechanism of GaAs Nanowires by Molecular Beam Epitaxy. Phys. Rev. B.

[26] Dubrovskii V.G., Fontcuberta i Morral A., Dayeh S.A., Jagadish C. (2015). Theory of VLS growth of compound semiconductors. Semiconductors and Semimetals.

[27] So H., Pan D., Li L., Zhao J. (2017). Foreign-Catalyst-Free Growth of InAs/InSb Axial Heterostructure Nanowires on Si (111) by Molecular-Beam Epitaxy. Nanotechnology.

[28] Pozuelo M., Zhou H., Lin S., Lipman S.A., Goorsky M.S., Hicks R.F., Kodambaka S. (2011). Self-Catalyzed Growth of InP/InSb Axial Nanowire Heterostructures. J. Cryst. Growth.

[29] Priante G., Ambrosini S., Dubrovskii V.G., Franciosi A., Rubini S. (2013). Stopping and Resuming at Will the Growth of GaAs Nanowires. Cryst. Growth Des..

[30] Yuan Y., Randall Lee T., Bracco G., Holst B. (2013). Contact angle and wetting properties. Surface Science Techniques.

[31] Panciera F., Baraissov Z., Patriarche G., Dubrovskii V.G., Glas F., Travers L., Mirsaidov U., Harmand J.C. (2020). Phase selection in self-catalyzed GaAs nanowires. Nano Lett..

[32] Glas F., Ramdani M.R., Patriarche G., Harmand J.C. (2013). Predictive Modeling of Self-Catalyzed III-V Nanowire Growth. Phys. Rev. B.

[33] Kim W., Dubrovskii V.G., Vukajlovic-Plestina J., Tütüncüoglu G., Francaviglia L., Güniat L., Potts H., Friedl M., Leran J.B., Fontcuberta I. (2018). Bistability of Contact Angle and Its Role in Achieving Quantum-Thin Self-Assisted GaAs Nanowires. Nano Lett..

[34] Dubrovskii V.G., Xu T., Álvarez A.D., Plissard S.R., Caroff P., Glas F., Grandidier B. (2015). Self-Equilibration of the Diameter of Ga-Catalyzed GaAs Nanowires. Nano Lett..

[35] Leshchenko E.D., Kuyanov P., LaPierre R.R., Dubrovskii V.G. (2018). Tuning the morphology of self-assisted GaP nanowires. Nanotechnology.

[36] Dubrovskii V.G., Avouris P., Bhushan B., Klitzing K.V., Sakaki H., Wiesendanger R. (2014). Nucleation Theory and Growth of Nanostructures. NanoScience and Technology.

[37] Dubrovskii V.G., Sibirev N.V., Berdnikov Y., Gomes U.P., Ercolani D., Zannier V., Sorba L. (2016). Length Distributions of Au-Catalyzed and In-Catalyzed InAs Nanowires. Nanotechnology.

[38] Dubrovskii V.G., Berdnikov Y., Schmidtbauer J., Borg M., Storm K., Deppert K., Johansson J. (2016). Length distributions of nanowires growing by surface diffusion. Cryst. Growth Des..

[39] Tersoff J. (2015). Stable Self-Catalyzed Growth of III-V Nanowires. Nano Lett..

[40] Glas F. (2010). Vapor Fluxes on the Apical Droplet during Nanowire Growth by Molecular Beam Epitaxy. Phys. Status Solidi B.

[41] Dubrovskii V.G. (2017). Development of Growth Theory for Vapor-Liquid-Solid Nanowires: Contact Angle, Truncated Facets, and Crystal Phase. Cryst. Growth Des..

[42] Dubrovskii V.G. (2019). Stabilization of the Morphology and Crystal Phase in Ensembles of Self-Catalyzed GaAs Nanowires. Phys. Status Solidi Rapid Res. Lett..

