# Supplementary material for: Growth of Self-Catalyzed InAs/InSb Axial Heterostructured Nanowires: Experiment and Theory
Source: Nanomaterials (Basel). 2020 Mar 10;10(3):494. doi: 10.3390/nano10030494 (PMC7153585; doi:10.3390/nano10030494)
Supplement: Supplementary file 1 [file nanomaterials-10-00494-s001.pdf]

nanomaterials

MDPI

Article

Growth of self-catalyzed InAs/InSb axial heterostructured nanowires: experiment and theory

Omer Arif [1], Valentina Zannier [1,*], Vladimir G. Dubrovskii [2], Igor V. Shtrom [3], Francesca Rossi [4,*], Fabio Beltram [1] and Lucia Sorba [1]

1 NEST, Istituto Nanoscienze—CNR and Scuola Normale Superiore, Piazza San Silvestro 12, I-56127 Pisa, Italy; omer.arif@sns.it (O.A.); fabio.beltram@sns.it (F.B.); lucia.sorba@nano.cnr.it (L.S.)

2 School of Photonics, ITMO University, Kronverkskiy pr. 49, 197101 St. Petersburg, Russia; dubrovskii@mail.ioffe.ru

3 The Faculty of Physics, St. Petersburg State University, Universitetskaya Emb. 13B, 199034, St. Petersburg, Russia; i.shtorm@spbu.ru

4 IMEM—CNR, Parco Area delle Scienze 37/A, I-43124 Parma, Italy

* Correspondence: valentina.zannier@nano.cnr.it (V.Z.); francesca.rossi@imem.cnr.it (F.R.); Tel.: +39-050-509-123(474) (V.Z.); +39-0521-269-221 (F.R.)

Supplementary material

S1. Measured parameters of the NWs of all sample series

S2. Vapor-solid growth of InSb

S3. High-resolution transmission electron microscopy analysis

S4. X-ray energy dispersive spectroscopy data on the catalyst droplet

S5. ZB crystal phase of InSb segments

S6. Cooling down experiment

S7. InSb length at short growth times

S1. Measured parameters of the NWs of all sample series

In order to measure the nanoparticle (NP) height H and base radius R_d, the InSb segment length L and maximum diameter D, the nanowires (NWs) were mechanically transferred from as grown substrates onto Si substrates and 90° projection images were taken with scanning electron microscopy (SEM). We measured about 30 NWs for each sample and we calculated the NP aspect ratio (H/R_d) and contact angle (β). The following tables show the average values with the errors representing the standard deviation.

Table S1.1. Measured and calculated geometrical parameters for the time series reported in Figure 1 (a) of the main text: InSb segments grown under line pressures of F_{In} = 0.2 Torr and F_{Sb} = 0.35 Torr.

InSb growth time (min)	R_d (nm)	H (nm)	NP aspect ratio (H/R_d)	β (Degree)	D (nm)	L (nm)
10	26 ± 3	18 ± 3	0.69 ± 0.20	70 ± 7	79 ± 3	65 ± 5
15	29 ± 2	29 ± 3	1 ± 0.12	91 ± 5	70 ± 4	78 ± 6
20	41 ± 2	44 ± 3	1.07 ± 0.08	94 ± 4	88 ± 3	91 ± 4
30	45 ± 4	58 ± 8	1.28 ± 0.16	104 ± 8	99 ± 12	88 ± 10
45	54 ± 2	70 ± 4	1.29 ± 0.06	106 ± 4	112 ± 5	110 ± 10

60	66 ± 3	82 ± 5	1.24 ± 0.07	102 ± 4	139 ± 4	142 ± 12
90	70±3	90 ± 8	1.28 ± 0.09	104±5	145 ± 7	160 ± 7
120	90 ± 6	94 ± 11	1.04 ± 0.13	93 ± 3	201 ± 13	321 ± 17
180	121 ± 4	142 ± 20	1.17 ± 0.14	99 ± 9	244 ± 11	413 ± 40

Table S1.2. Measured and calculated geometrical parameters for the time series reported in Figure 1 (e) of the main text: InSb segments grown under line pressures of F_{In} = 0.2 Torr and F_{Sb} = 0.70 Torr.

InSb growth time (min)	R_d (nm)	H (nm)	NP aspect ratio (H/R_d)	β (Degree)	D (nm)	L (nm)
30	39 ± 2	34 ± 4	0.87 ± 0.12	82 ± 5	107 ± 8	135 ± 7
45	39 ± 2	36 ± 6	0.92 ± 0.17	80 ± 4	123 ± 7	200 ± 18
60	45 ± 3	35 ± 3	0.77 ± 0.10	79 ± 4	140 ± 4	266 ± 10

Table S1.3. Measured and calculated geometrical parameters for the samples reported in Figure 2 (a) of the main text: InAs/InSb NWs obtained keeping F_{In} at 0.2 Torr and varying F_{Sb} from 0.35 to 0.80 Torr.

F_{Sb} (Torr)	R_d (nm)	H (nm)	NP aspect ratio (H/R_d)	β (Degree)	D (nm)	L (nm)
0.35	66 ± 3	82 ± 5	1.24 ± 0.07	102 ± 4	139 ± 4	142 ± 12
0.40	65 ± 3	75 ± 6	1.15 ± 0.09	98 ± 5	139 ± 9	171 ± 20
0.45	62 ± 5	61 ± 6	0.98 ± 0.12	89 ± 3	147 ± 6	203 ± 14
0.50	61 ± 2	59 ± 3	0.96 ± 0.06	88 ± 2	148 ± 8	204 ± 9
0.55	54 ± 2	48 ± 3	0.88 ± 0.07	84 ± 3	146 ± 3	243 ± 10
0.65	53 ± 2	42 ± 3	0.79 ± 0.08	78 ± 3	150 ± 3	258 ± 20
0.70	45 ± 3	35 ± 3	0.77 ± 0.10	79 ± 4	140 ± 4	266 ± 10
0.80	42 ± 2	34 ± 4	0.80 ± 0.12	79 ± 5	147 ± 4	279 ± 15

Table S1.4. Measured and calculated geometrical parameters for the samples reported in Figure 3 (a) of the main text: InAs/InSb NWs obtained keeping F_{Sb} at 0.35 Torr and varying F_{In} from 0.2 to 0.65 Torr.

F_{In} (Torr)	R_d (nm)	H (nm)	NP aspect ratio (H/R_d)	β (Degree)	D (nm)	L (nm)
0.2	66 ± 3	82 ± 5	1.24 ± 0.07	102 ± 4	139 ± 4	142 ± 12
0.25	73 ± 3	101 ± 5	1.38 ± 0.06	108 ± 2	151 ± 6	147 ± 7
0.30	72 ± 9	100 ± 12	1.38 ± 0.17	108 ± 3	152 ± 17	154 ± 17
0.35	77 ± 11	116 ± 16	1.50 ± 0.19	113 ± 4	156 ± 19	153 ± 20
0.40	87 ± 7	134 ± 20	1.54 ± 0.16	115 ± 2	175 ± 12	160 ± 10
0.45	96 ± 7	161 ± 10	1.67 ± 0.09	118 ± 3	195 ± 20	160 ± 12
0.55	102 ± 7	190 ± 13	1.86 ± 0.09	123 ± 4	204 ± 13	156 ± 8
0.65	117 ± 7	239 ± 8	2.04 ± 0.06	128 ± 2	225 ± 9	146 ± 17

S2. Vapor-solid growth of InSb

When F_{Sb} is increased to 0.9 Torr, a transition from the VLS growth to the catalyst-free vapor-solid (VS) mode (without any In droplet) occurs, and no more axial growth of InSb is observed. Instead, InSb starts forming a shell around the InAs stem, as we can see in the SEM image S2.1.

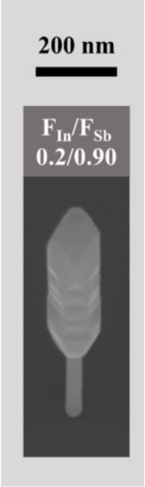

Figure S2.1. 45° tilted **S**EM image of a NW obtained with F_{In}/F_{Sb} = 0.2/0.90 and t = 60 min, the shell-like growth of InSb is clearly visible around InAs stem.

S3. High-resolution transmission electron microscopy analysis

We performed TEM analysis of the InAs/InSb NWs grown under different precursor line pressure for the same InSb growth time of 60 min. Figure S3.1 shows HR-TEM images of InAs/InSb NWs grown under highly In-rich conditions (F_{In}/F_{Sb} = 0.65/0.35). Similarly, to the NWs grown with much lower F_{In}/F_{Sb} ratio (Figure 4 of the main text), the crystal structure of the InSb segment is pure ZB with only a few stacking faults followed by a thin WZ portion close to the NW tip.

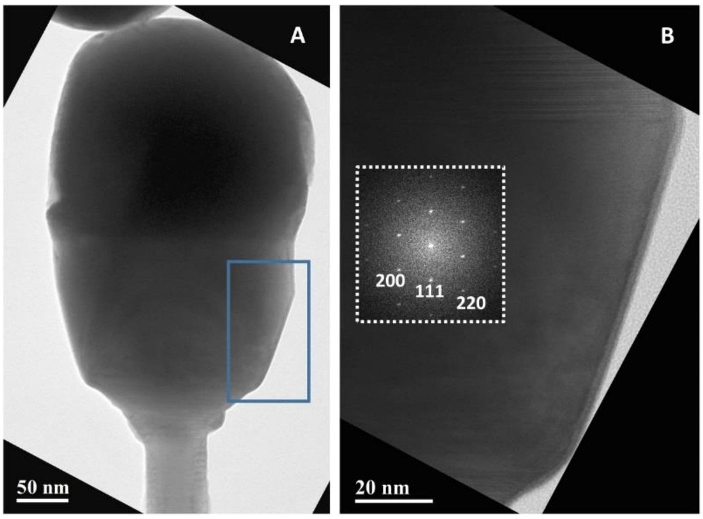

Figure S3.1. HR-TEM image of a NW obtained with F_{In}/F_{Sb} = 0.65/0.35 and t = 60 min: the whole NW (a) and the selected section (b) framed in (a). The inset in (b) shows the fast Fourier transform (FFT) of the image.

S4. X-ray energy dispersive spectroscopy data on the catalyst droplet

The droplet composition for NWs taken from three different samples (grown under different In and Sb line pressure) were measured by energy dispersive X-ray spectroscopy (EDX), with the results summarized in the Table S4.1.

Table S4.1. Droplet compositions measured by EDX

Growth parameters		Composition of the Droplet	
InSb growth time (min)	Precursor line pressures (F_{In}/F_{Sb})	In (%)	Sb (%)
60	0.2/0.35	99.5	0.5
60	0.2/0.7	99.4	0.6
60	0.65/0.35	99.6	0.4

S5. ZB crystal phase of InSb segments

Here, we briefly discuss the crystal phase purity of InSb NW sections in the ZB structure from the surface energy point of view (see Figure 5 of the main text for the model parameters). We note that the difference between planar solid-vapor and solid-liquid facets for InSb $\gamma_{SV} - \gamma_{SL} =$ 0.0936 J/m^2 is quite small, corresponding to the Young's angle which is close to 90°. The Glas condition for the WZ phase formation [1], is given by $\gamma_{SV}^{l} - \gamma_{SL}^{l} - \gamma_{LV} \sin\beta < 0$, where γ_{SV}^{l} and γ_{SL}^{l} are the surface energies of the corresponding vertical facets. Assuming a small $\gamma_{SV}^{l} - \gamma_{SL}^{l}$, as for planar facets, the WZ phase seems to be always enabled around $\beta =$ 90°, as in our case. In fact, this observation was the original argument of Ref. [1], for the prevalence of the WZ phase in VLS III-V NWs. However, these considerations apply to vertical corner facets wetted by the droplet. It was then noticed that III-V NWs may grow with a truncated corner facets which makes nucleation of two-dimensional (2D) islands at the TPL improbable and hence the crystal phase of such truncated NWs should be ZB [2, 3]. Therefore, being the ZB phase very predominant in VLS InSb NWs in almost all cases, we speculate that InSb islands always nucleate with truncated lateral facets, as suggested in Ref. [4]. A more detailed analysis of the growth interface of InSb NWs grown by self-catalyzed CBE will be presented elsewhere.

S6. Cooling down experiment

In order to study the effect of the cooling process on the NW morphology and the droplet shape, we have grown two samples using the same parameters (F_{In}/F_{Sb} = 0.2/0.35 and 60 min growth time) but with different growth terminations. In the first case, the sample was cooled down to 150 °C in 3 min under TDMASb line pressure (as for all the other samples reported in the main text, linearly decreasing the line pressure from 0.35 Torr to 0 Torr), while in the second case it was cooled down without any precursor flux. Scanning electron microscopy (SEM) micrographs of the NWs obtained are shown in Figure S6.1. We measured the length and diameter of InSb segments and the contact angle of the droplets of ~ 30 NWs from each sample, following the procedure described in the main text. We found no difference between the two samples in terms of the NW length, diameter and contact angle of the droplet. We obtained L = 180 ± 10 nm, D = 162 ± 3 nm, and β = 104°± 2° for the sample cooled down under TDMASb line pressure; and L = 190 ± 10 nm, D = 157 ± 3 nm, and β = 102° ± 2° for the sample cooled down without any flux. Therefore, we concluded that the cooling down process does not affect the morphology of the InSb segment and the In droplet shape. This is reasonable considering that the

axial growth will immediately decrease and probably stop during the cooling down step due to the lower temperature and the lower amount of Sb atoms available in vapor phase [5].

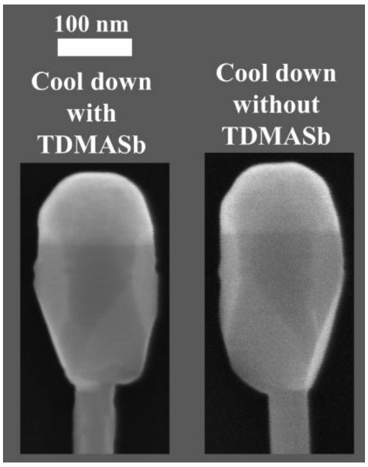

Figure S6.1. SEM images of InSb/InAs NW grown under F_{In} = 0.2 Torr and F_{Sb} = 0.35 Torr for 60 min and cooled down under TDMASb flux (left panel) and without any flux (right panel).

S7. InSb length at short growth times

Here we discuss one of the possible explanations of the super-linear axial InSb growth rate for short growth times (< 30 min) observed in the series of samples grown using F_{In} = 0.2 Torr and F_{Sb} = 0.35 Torr (see Figure 1 (a) and (c) of the main text). As known from previously reported TEM analysis of the catalyst-free InAs NWs [6], the NW tip is not perfectly flat, showing instead some inclined facets, resulting in an tapered tip terminating with the flat (111) top facet. The tapered InAs tip shape is also visible in the EDX map of our InAs/InSb NWs (see figure 4 (a) of the main text). However, when we perform SEM imaging if the InAs/InSb NWs, the tapered InAs tip is not visible anymore, suggesting that InSb growth occurs also on the inclined facets of the InAs stem, burying the tapered InAs tip. Indeed, when we measure the InSb segment length (L), we take the InAs/InSb interface as the point at which we see the increase of the NW diameter. Therefore, L can differ from the actual InSb axial segment length (L^*), and this will result in an overestimation of the segment length, which is more relevant for short growth times. Figure S7.1 schematically explains this effect: the measured parameter L is given by the actual axial segment length L^* plus the height of the tapered InAs tip (h). Since the latter is around 30-40 nm (as measured from the EDX maps), the overestimation of L is more relevant for short growth times ($t \leq$ 30 min).

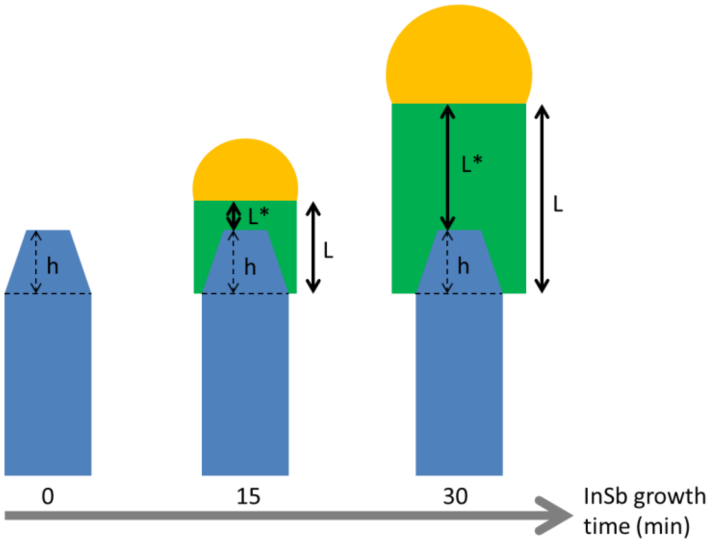

Figure S7.1. Schematic representation of the early stages of InSb growth. The measured InSb segment length (*L*) is overestimated compared to the real axial segment length (L^*) due to the InSb radial growth on the inclined facets of the InAs stem tip that results in a wrong InAs/InSb horizontal interface positioning.

References

1. Glas, F.; Harmand, J. C.; Patriarche, G. Why Does Wurtzite Form in Nanowires of III-V Zinc Blende Semiconductors? *Phys. Rev. Lett.* **2007**, *99*, 146101–146104. https://link.aps.org/doi/10.1103/PhysRevLett.99.146101.
2. Wen, C. Y.; Tersoff, J.; Hillerich, K.; Reuter, M. C.; Park, J. H.; Kodambaka, S.; Stach, E. A.; Ross, F. M. Periodically Changing Morphology of the Growth Interface in Si, Ge, and GaP Nanowires. *Phys. Rev. Lett.* **2011**, *107*, 025503–025506. https://link.aps.org/doi/10.1103/PhysRevLett.107.025503.
3. Jacobsson, D.; Panciera, F.; Tersoff, J.; Reuter, M. C.; Lehmann, S.; Hofmann, S.; Dick, K. A.; Ross, F. M. Interface Dynamics and Crystal Phase Switching in GaAs Nanowires. *Nature* **2016**, *531*, 317–322. https://doi.org/10.1038/nature17148.
4. Dubrovskii, V. G.; Sibirev, N. V.; Halder, N. N.; Ritter, D. Classification of the Morphologies and Related Crystal Phases of III–V Nanowires Based on the Surface Energy Analysis. *J. Phys. Chem. C* **2019**, *123*, 18693–18701. https://doi.org/10.1021/acs.jpcc.9b05028.
5. Salim, S.; Lim, C. K.; Jensen, F. Gas-phase Decomposition Reactions of Tris(dimethylamino)phosphine, -arsine, and -stibine reagents. *Chem. Mater.* **1995**, *7*, 507-516. https://doi.org/10.1021/cm00051a011.
6. Gomes, U. P.; Ercolani, D.; Sibirev, N. V.; Gemmi, M.; Dubrovskii, V. G.; Beltram, F.; Sorba, L. Catalyst-Free Growth of InAs Nanowires on Si (111) by CBE. *Nanotechnology* **2015**, *26*, 415604-415614. doi:10.1088/0957-4484/26/41/415604.
